# Amplification of elliptically polarized sub-femtosecond pulses in neon-like X-ray laser modulated by an IR field

**DOI:** 10.1038/s41598-022-09701-z

**Published:** 2022-04-13

**Authors:** I. R. Khairulin, V. A. Antonov, M. Yu. Ryabikin, M. A. Berrill, V. N. Shlyaptsev, J. J. Rocca, Olga Kocharovskaya

**Affiliations:** 1 Institute of Applied Physics of the Russian Academy of Sciences, 46 Ulyanov Street, Nizhny Novgorod, Russia 603950; 2 Lobachevsky State University of Nizhny Novgorod, 23 Prospekt Gagarina, Nizhny Novgorod, Russia 603950; 3 Department of Electrical and Computer Engineering, Colorado State University, Fort Collins, CO 80523 USA; 4 Department of Physics, Colorado State University, Fort Collins, CO 80523 USA; 5 Department of Physics and Astronomy and Institute for Quantum Science and Engineering, Texas A&M University, 578 University Drive, College Station, TX 77843-4242 USA

**Keywords:** Ultrafast photonics, X-rays

## Abstract

Amplification of attosecond pulses produced via high harmonic generation is a formidable problem since none of the amplifiers can support the corresponding PHz bandwidth. Producing the well defined polarization state common for a set of harmonics required for formation of the circularly/elliptically polarized attosecond pulses (which are on demand for dynamical imaging and coherent control of the spin flip processes) is another big challenge. In this work we show how both problems can be tackled simultaneously on the basis of the same platform, namely, the plasma-based X-ray amplifier whose resonant transition frequency is modulated by an infrared field.

## Introduction

In recent years, considerable attention has been paid to the generation of elliptically and circularly polarized high-order harmonics (HHs) of optical radiation. The interest in this problem is due to the possibility to use an elliptically polarized radiation of extreme ultraviolet (XUV) and X-ray ranges for probing the magnetic^1,2^ and chiral^3–5^ media and processes in them^6,7^, the development of spintronics^8^, etc.

Unfortunately, the straightforward approach, based on generation of the elliptically polarized HHs in a gas by elliptically polarized laser field of the fundamental frequency, is extremely inefficient because an ellipticity of the laser field leads to deviation of an accelerated electron from the straight pathway to the parent ion, greatly reducing the probability of recombination and hence suppressing high-harmonic generation (HHG)^9–12^.

In order to overcome these limitations, several approaches were suggested, including (i) the resonant enhancement of elliptically polarized harmonics^13,14^, (ii) conversion of linearly polarized harmonics to elliptically polarized ones via phase-shifting optics^15,16^, (iii) HHG from molecules, which often show a higher efficiency compared with atoms, especially for pump lasers with elliptical polarization^17,18^, and can lead to efficient generation of elliptically polarized harmonics in the case of aligned molecules^19,20^, as well as the use of (iv) cross-linearly-polarized two-color laser fields^21,22^, and (v) bielliptic^23^ or bicircular^24^ fields, which have led to the demonstration of the first bright tabletop soft X-ray source for magnetic circular dichroism measurements^25^. However, neither of them allows for the generation of sub-femtosecond XUV pulses with sufficiently high energy, high ellipticity and a well defined polarization state common for the harmonics of different orders. An amplification of 32.8 nm XUV radiation with circular polarization was experimentally shown in nickel-like Kr^8+^ active medium of a plasma-based X-ray laser^26^, but, in this case, only a single resonant harmonic was amplified. It is worth to point out that amplification of the attosecond pulses is itself a formidable problem since none of the existing amplifiers can support the corresponding PHz bandwidth.

In this work we present the new concept of using plasma-based X-ray lasers for (a) amplification of the sub-femtosecond XUV radiation pulses with arbitrary elliptical polarization produced via HHG, and (b) increasing ellipticity of this radiation. Both possibilities are provided by coherent dressing of the neon-like active medium of an X-ray laser with an intense infrared (IR) laser field. It is closely related to our recent work^27^, which has shown the possibility to amplify a set of linearly polarized HHs in a hydrogenlike active medium of a plasma-based X-ray laser dressed by a replica of a fundamental-frequency IR field with the same linear polarization. However, in the hydrogenlike medium the amplification of elliptically or circularly polarized HHs is prohibited, since the redistribution of the gain to the frequencies of harmonics is caused by the IR-field-induced sub-laser-cycle linear Stark effect and occurs only for XUV/X-rays with the same linear polarization as that of the modulating field, whereas for radiation with orthogonal polarization the gain remains localized at the single resonance frequency. Here we show that this limitation can be circumvented using a neon-like active medium, where the IR field results in the sub-laser-cycle space–time variation of energies of the resonant states of the ions due to the quadratic Stark effect, which provides the gain redistribution to combination frequencies for both polarization components of the XUV/X-ray field and makes possible the amplification of a set of HHs with arbitrary elliptical polarization.

### Theoretical model

Below we consider the active medium of a plasma-based X-ray laser with inversion at the 3*p*^1^S_0_ ↔ 3*s*^1^P_1_ transition of neon-like Ti^12+^ ions with the unperturbed resonance wavelength of 32.6 nm^28–34^. The plasma channel can be produced by a sequential illumination of the polished titanium metal surface with two pulses focused into a line. The first pulse produces the plasma and the second one provides a population inversion via collisional pumping. The key element in making a nearly uniform channel is using a grazing incidence for the pumping pulse^31,34^. Alternatively, the plasma can be created by a discharge and pumped with an intense ultrashort laser pulse propagating in a capillary plasma waveguide, similarly to^35^. The upper lasing energy level is nondegenerate and corresponds to the state |1〉 with the total momentum *J* = 0. The lower energy level is triply degenerate and corresponds to the |2〉, |3〉, and |4〉 states with *J* = 1 and the momentum projection on the quantization axis *M* = 0, *M* = 1, and *M* = − 1, respectively.

**Figure 1:**
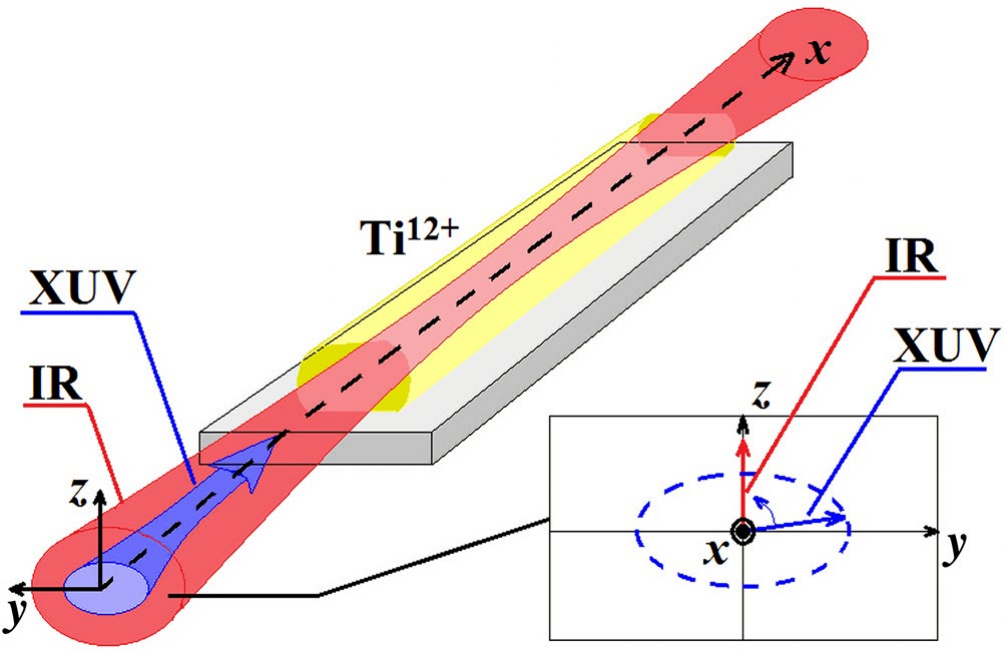
A sketch of the model considered in the paper. The pre-created plasma active medium (yellow truncated cylinder) is irradiated by a combination of a strong IR field (red beam) and a set of its high-order harmonics (the XUV radiation, violet beam), which overlap in space and time and co-propagate along the axis of the plasma channel (*x*-axis). The IR field is linearly polarized along *z*-axis, while the XUV beam has an arbitrary elliptical polarization in *yz*-plane.

Further, we assume that the pre-created active medium has the form of a thin cylinder oriented along the *x* axis. It is irradiated with an IR field propagating along the same x axis and polarized along the *z* axis (red beam in Fig. 1). In practice, different designs for the IR coherent drive injection can be implemented (for instance, based on channeling by a capillary waveguide or grazing incidence) as the phenomenon of the amplification of the elliptically polarized harmonics described below is due solely to the time modulation of energies of the resonant states of the ions and does not imply the phase-matching of the IR field with the amplified XUV radiation. We consider the IR field of the form:

1 $$\vec{E}_{IR} \left( {x,t} \right) = \vec{z}_{0} E_{M} \cos \left[ {\Omega \left( {t - {{x\sqrt {\varepsilon_{pl}^{(IR)} } } \mathord{\left/ {\vphantom {{x\sqrt {\varepsilon_{pl}^{(IR)} } } c}} \right. \kern-\nulldelimiterspace} c}} \right)} \right]$$

**Figure 2:**
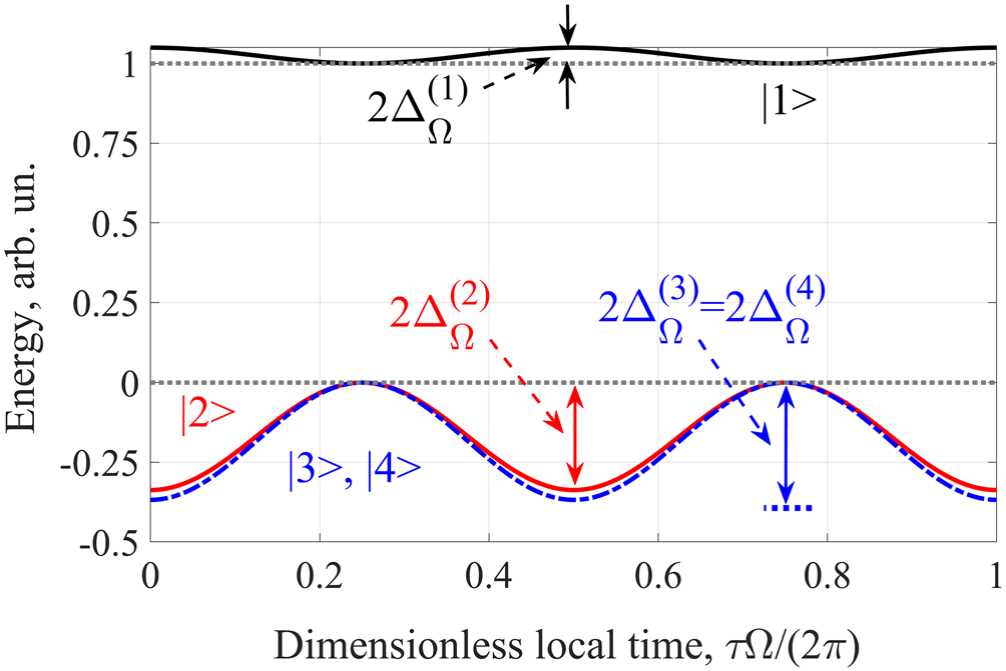
Stark shift of the relevant energy levels of Ti^12+^ ions calculated according to Eq. (2) under the action of the IR field with intensity 8.26 × 10^16^ W/cm^2^. Black and red solid curves correspond to the states |1〉 = |3*p*^1^S_0_, J = 0, M = 0〉 and |2〉 = |3*s*^1^P_1_, J = 1, M = 0〉, respectively. Blue dash-dotted curve shows the degenerate energy level, which corresponds to the states |3〉 = |3*s*^1^P_1_, J = 1, M = 1〉 and |4〉 = |3*s*^1^P_1_, J = 1, M = − 1〉. Grey dotted horizontal lines are the position of the upper and lower lasing energy levels in the absence of the IR field.

Here $\vec{z}_{0}$ is the unit vector along the *z* axis, $E_{M}$ and $\Omega$ are the amplitude and frequency of the IR field, *c* is the speed of light in vacuum, $\varepsilon_{pl}^{(IR)} = 1 - {{\omega_{pl}^{2} } \mathord{\left/ {\vphantom {{\omega_{pl}^{2} } {\Omega^{2} }}} \right. \kern-\nulldelimiterspace} {\Omega^{2} }}$ is the dielectric constant of the plasma for the IR field, $\omega_{pl} = \sqrt {{{4\pi N_{e} e^{2} } \mathord{\left/ {\vphantom {{4\pi N_{e} e^{2} } {m_{e} }}} \right. \kern-\nulldelimiterspace} {m_{e} }}}$ is the electron plasma frequency, $N_{e}$ is the concentration of free electrons in the plasma, *e* and $m_{e}$ are the charge and mass of the electron, respectively. In Eq. (1), the pulse duration of the IR field is assumed to be significantly longer than the duration of all the processes under study, which allows us to consider it as a monochromatic one. Both the frequency of the IR field and its Rabi frequencies on the electric-dipole-allowed transitions from the states |1〉–|4〉 in Ti^12+^ ions are much lower than the frequencies of these transitions. As a result, the major effect of the IR field on the |1〉–|4〉 states is a shift of the corresponding energy levels due to the quadratic Stark effect, see Fig. 2. In this case, the position of the *i-*th energy level (*i* = 1, 2, 3, 4) is determined by^36^

2 $${\rm E}_{i} \left( {x,t} \right) = {\rm E}_{i}^{\left( 0 \right)} + \frac{1}{2}\sum\limits_{k \ne i} {\frac{{\left| {d_{ki}^{(z)} } \right|^{2} E_{M}^{2} }}{{\hbar \omega_{ik} }}\left\{ {1 + \cos \left[ {2\Omega \left( {t - \frac{{\sqrt {\varepsilon_{pl}^{(IR)} } }}{c}x} \right)} \right]} \right\}} ,$$

where ${\rm E}_{i}^{\left( 0 \right)}$ is the unperturbed energy value, $\omega_{ik}$ is the unperturbed frequency of the transition from the |*i*〉 state to the |*k*〉 state, $\hbar$ is Planck's constant, and $d_{ki}^{(z)}$ is the projection of the dipole moment of a given transition on the *z* axis. The summation in (2) is carried out over all the relevant states of the field-free ion (including the states |1〉–|4〉)^37^. We further introduce the notations $\Delta_{\rm E}^{\left( i \right)} = \sum\limits_{k \ne i} {{{\left( {\left| {d_{ki}^{(z)} } \right|E_{M} } \right)^{2} } \mathord{\left/ {\vphantom {{\left( {\left| {d_{ki}^{(z)} } \right|E_{M} } \right)^{2} } {\left( {2\hbar \omega_{ik} } \right)}}} \right. \kern-\nulldelimiterspace} {\left( {2\hbar \omega_{ik} } \right)}}}$ for the amplitude of the energy shift of the *i-*th state and $\Delta_{\Omega }^{{\left( {ij} \right)}} = {{\left( {\Delta_{\rm E}^{\left( i \right)} - \Delta_{\rm E}^{\left( j \right)} } \right)} \mathord{\left/ {\vphantom {{\left( {\Delta_{\rm E}^{\left( i \right)} - \Delta_{\rm E}^{\left( j \right)} } \right)} \hbar }} \right. \kern-\nulldelimiterspace} \hbar }$ for the amplitude of the frequency change of the |*i*〉 ↔|*j*〉 transition, where *i*, *j* = 1, 2, 3, 4.

In addition to the IR field, the active medium is irradiated with a set of its odd HHs (violet beam in Fig. 1) of orders ranging from 2(*q-k*_min_) + 1 to 2(*q* + *k*_max_) + 1, which at the input to the medium, *x* = 0, has the form

3 $$\vec{E}^{(inc)} (t) = \frac{1}{2}\sum\limits_{{k = k_{\min } }}^{{k_{\max } }} {\left( {\vec{z}_{0} \tilde{E}_{z,\,inc}^{{{\kern 1pt} (k)}} (t) + \vec{y}_{0} \tilde{E}_{y,\,inc}^{{{\kern 1pt} (k)}} (t)} \right)} \exp \left[ { - i\left( {\omega + 2k\Omega } \right){\kern 1pt} t} \right] + {\text{c.c}}.,$$

where $\vec{y}_{0}$ is the unit vector along the *y* axis, $\omega = \left( {2q + 1} \right)\Omega$ is the carrier frequency of the HH field, *q* is an integer, $\tilde{E}_{z,\,inc}^{{{\kern 1pt} (k)}} (t)$ and $\tilde{E}_{y,\,inc}^{{{\kern 1pt} (k)}} (t)$ are the slowly varying amplitudes of *z-* and *y-*polarization components of the field of the “*k-*th” harmonic with the frequency $\omega_{k} = \omega + 2k\Omega$, and c.c. denotes a complex conjugate.

The coupled space–time evolution of the HH field and the quantum state of Ti^12+^ ions is described by the one-dimensional wave equation and the density-matrix equations (see Supplement 1) with the space–time dependent frequencies of quantum transitions between the |1〉, |2〉, |3〉, and |4〉 states, which can be represented as

4 $$\left\{ \begin{gathered} \omega_{12} \left( {\tau ,x} \right) = \overline{\omega }_{tr}^{(z)} + \Delta_{\Omega }^{\left( z \right)} \cos \left[ {2\left( {\Omega \tau + \Delta Kx} \right)} \right], \hfill \\ \omega_{13} \left( {\tau ,x} \right) = \overline{\omega }_{tr}^{(y)} + \Delta_{\Omega }^{\left( y \right)} \cos \left[ {2\left( {\Omega \tau + \Delta Kx} \right)} \right], \hfill \\ \omega_{23} \left( {\tau ,x} \right) = \left( {\Delta_{\Omega }^{\left( z \right)} - \Delta_{\Omega }^{\left( y \right)} } \right)\left\{ {1 + \cos \left[ {2\left( {\Omega \tau + \Delta Kx} \right)} \right]} \right\}, \hfill \\ \end{gathered} \right.$$

$\omega_{14} \left( {\tau ,x} \right) = \omega_{13} \left( {\tau ,x} \right),$  $\omega_{24} \left( {\tau ,x} \right) = \omega_{23} \left( {\tau ,x} \right),$ and $\omega_{34} \left( {\tau ,x} \right) = 0,$ where $\tau = t - {{x\sqrt {\varepsilon_{pl}^{(XUV)} } } \mathord{\left/ {\vphantom {{x\sqrt {\varepsilon_{pl}^{(XUV)} } } c}} \right. \kern-\nulldelimiterspace} c}$ is the local time in the reference frame moving along the *x* axis with the phase velocity of the HH radiation in the plasma, $\varepsilon_{pl}^{(XUV)} = 1 - {{\omega_{pl}^{2} } \mathord{\left/ {\vphantom {{\omega_{pl}^{2} } {\omega^{2} }}} \right. \kern-\nulldelimiterspace} {\omega^{2} }}$ is the dielectric constant of the plasma for the HH radiation ($\omega \gg \Omega$, so that $\varepsilon_{pl}^{(XUV)} \simeq 1$), $\Delta K = {{\Omega \left( {\sqrt {\varepsilon_{pl}^{(XUV)} } - \sqrt {\varepsilon_{pl}^{(IR)} } } \right)} \mathord{\left/ {\vphantom {{\Omega \left( {\sqrt {\varepsilon_{pl}^{(XUV)} } - \sqrt {\varepsilon_{pl}^{(IR)} } } \right)} c}} \right. \kern-\nulldelimiterspace} c}$ is an addition to the wave number of the IR field due to the difference between its phase velocity and the HH radiation phase velocity, $\overline{\omega }_{tr}^{(z)} \equiv {{\left( {{\rm E}_{1}^{\left( 0 \right)} - {\rm E}_{2}^{\left( 0 \right)} } \right)} \mathord{\left/ {\vphantom {{\left( {{\rm E}_{1}^{\left( 0 \right)} - {\rm E}_{2}^{\left( 0 \right)} } \right)} \hbar }} \right. \kern-\nulldelimiterspace} \hbar } + \Delta_{\Omega }^{\left( z \right)} \;$ is the time-averaged |1〉 ↔ |2〉 transition frequency, $\overline{\omega }_{tr}^{(y)} \equiv {{\left( {{\rm E}_{1}^{\left( 0 \right)} - {\rm E}_{3}^{\left( 0 \right)} } \right)} \mathord{\left/ {\vphantom {{\left( {{\rm E}_{1}^{\left( 0 \right)} - {\rm E}_{3}^{\left( 0 \right)} } \right)} \hbar }} \right. \kern-\nulldelimiterspace} \hbar } + \Delta_{\Omega }^{\left( y \right)} =$ ${{\left( {{\rm E}_{1}^{\left( 0 \right)} - {\rm E}_{4}^{\left( 0 \right)} } \right)} \mathord{\left/ {\vphantom {{\left( {{\rm E}_{1}^{\left( 0 \right)} - {\rm E}_{4}^{\left( 0 \right)} } \right)} \hbar }} \right. \kern-\nulldelimiterspace} \hbar } + \Delta_{\Omega }^{\left( y \right)}$ is the time-averaged frequency of |1〉 ↔ |3〉 and |1〉 ↔ |4〉 transitions, $\Delta_{\Omega }^{\left( z \right)} \equiv \Delta_{\Omega }^{{\left( {12} \right)}}$, and $\Delta_{\Omega }^{\left( y \right)} \equiv \Delta_{\Omega }^{{\left( {13} \right)}} = \Delta_{\Omega }^{{\left( {14} \right)}}$. For neon-like ions, both the average values and the amplitudes of the frequency shift of the |1〉 ↔ |2〉 and |1〉 ↔ |3〉, |4〉 transitions interacting with *z*- and *y*-polarized components of XUV radiation are different: $\overline{\omega }_{tr}^{(z)} \ne \overline{\omega }_{tr}^{(y)}$ and $\Delta_{\Omega }^{\left( z \right)} \ne \Delta_{\Omega }^{\left( y \right)}$ as a result of different quadratic Stark shift of the states |2〉 and |3〉, |4〉.

The sub-IR-field-cycle modulation of the frequencies of transitions |1〉 ↔ |2〉 and |1〉 ↔ |3〉, |4〉 (4) leads to the appearance of the induced gain lines for each of the polarization components of the XUV radiation, which are separated from the time-averaged frequency of the corresponding transition by even multiples of the frequency of the IR field. In order to amplify a set of elliptically polarized harmonics, the gain lines for the *z*- and *y*-polarized components of the XUV radiation should overlap. This means that the gain spectra (combs of induced gain lines) for these polarization components should either coincide (which is not the case because of the different quadratic Stark shifts of the different lasing states of the ions), or be shifted with respect to each other by an even multiple of the frequency of the modulating IR field (which is also a half frequency separation between the adjacent harmonics). Mathematically, the latter condition corresponds to $\overline{\omega }_{tr}^{(y)} - \overline{\omega }_{tr}^{(z)} = 2\Omega p$, where *p* is an integer (which is equivalent to $\Delta_{\rm E}^{\left( 3 \right)} - \Delta_{\rm E}^{\left( 2 \right)} = 2\hbar \Omega {\kern 1pt} p$). In the paper we assume *p* = 1, which is the easiest case for the experimental implementation. This condition links the frequency of the IR field with its intensity. Indeed, the modulating field intensity governs quadratic Stark shift of the lasing energy levels and determines the difference $\overline{\omega }_{tr}^{(y)} - \overline{\omega }_{tr}^{(z)}$, while the frequency of the IR field determines the spacing between the adjacent gain lines. In more details it will be discussed elsewhere. If this condition is satisfied, and the carrier frequency of harmonics coincides with the time-averaged frequency of the transition |1〉 ↔ |2〉, $\omega = \overline{\omega }_{tr}^{(z)}$, the analytical solution for the amplitudes of *z-* and *y-* polarization components of the “*k*-th” harmonic field inside the medium takes the form (see Supplement 1 for details):

5a $$\left\{ \begin{gathered} \tilde{E}_{z}^{{{\kern 1pt} (k)}} \left( {x,\tau } \right) = E_{z,\,0}^{{{\kern 1pt} (k)}} \theta (\tau )\exp \left[ {g_{k}^{(z)} \left( {P_{\Omega }^{\left( z \right)} ,\tau } \right)x} \right], \hfill \\ g_{k}^{(z)} \left( {P_{\Omega }^{\left( z \right)} ,\tau } \right) = g_{total} J_{k}^{2} \left( {P_{\Omega }^{\left( z \right)} } \right)\left( {1 - e^{{ - \gamma_{tr} \tau }} } \right), \hfill \\ \end{gathered} \right.$$

5b $$\left\{ \begin{gathered} \tilde{E}_{y}^{{{\kern 1pt} (k)}} \left( {x,\tau } \right) = E_{y,\,0}^{{{\kern 1pt} (k)}} \theta (\tau )\exp \left[ {g_{k - p}^{(y)} \left( {P_{\Omega }^{\left( y \right)} ,\tau } \right)x} \right], \hfill \\ g_{k - p}^{(y)} \left( {P_{\Omega }^{\left( y \right)} ,\tau } \right) = g_{total} J_{k - p}^{2} \left( {P_{\Omega }^{\left( y \right)} } \right)\left( {1 - e^{{ - \gamma_{tr} \tau }} } \right), \hfill \\ \end{gathered} \right.$$

where $\theta (\tau )$ is the Heaviside unit step function, $P_{\Omega }^{\left( z \right)} = {{\Delta_{\Omega }^{\left( z \right)} } \mathord{\left/ {\vphantom {{\Delta_{\Omega }^{\left( z \right)} } {\left( {2\Omega } \right)}}} \right. \kern-\nulldelimiterspace} {\left( {2\Omega } \right)}}$ and $P_{\Omega }^{\left( y \right)} = {{\Delta_{\Omega }^{\left( y \right)} } \mathord{\left/ {\vphantom {{\Delta_{\Omega }^{\left( y \right)} } {\left( {2\Omega } \right)}}} \right. \kern-\nulldelimiterspace} {\left( {2\Omega } \right)}}$ are the IR field-induced frequency modulation indices of the |1〉 ↔ |2〉 and |1〉 ↔ |3〉, |4〉 transitions, respectively, $g_{k}^{(z)} \left( {P_{\Omega }^{\left( z \right)} ,\tau } \right)$ and $g_{k - p}^{(y)} \left( {P_{\Omega }^{\left( z \right)} ,\tau } \right)$ are the effective gain coefficients for *z-* and *y-* polarization components of the HH field, $g_{total}$ is the gain factor in the absence of the Stark effect, $J_{k} \left( x \right)$ is the Bessel function of the first kind of order *k*, and $\gamma_{tr}$ is the decoherence rate at the transitions |1〉 ↔ |2〉 and |1〉 ↔ |3〉, |4〉. The analytical solution (5a) and (5b) implies that during the considered time interval, the population differences at the inverted transitions are constant, the plasma is strongly dispersive for the modulating IR field so that there is no rescattering of HHs into each other, the spontaneous emission is negligible, and each harmonic in (3) is turned on instantly at $t = 0$ and then has a constant amplitude: $\tilde{E}_{z,\,inc}^{{{\kern 1pt} (k)}} (t) = \theta (t)E_{z,\,0}^{{{\kern 1pt} (k)}}$ and $\tilde{E}_{y,\,inc}^{{{\kern 1pt} (k)}} (t) = \theta (t)E_{y,\,0}^{{{\kern 1pt} (k)}}$, where $E_{z,\,0}^{{{\kern 1pt} (k)}}$ and $E_{y,\,0}^{{{\kern 1pt} (k)}}$ are complex numbers. In addition, the solution (5a) and (5b) implies an inertialess relationship between the resonant polarization of the medium and the XUV radiation (see^38^). The numerical results presented below were obtained without using any of the listed approximations.

As follows from Eqs. (5a) and (5b), the gain of the active medium is redistributed to multiple sidebands for both polarization components of the XUV field, and in the case $\overline{\omega }_{tr}^{(y)} - \overline{\omega }_{tr}^{(z)} = 2\Omega p$ the “*k-*th” induced gain line for *z-* polarization component of the XUV field overlaps with the gain line for *y-* polarization component numbered *k*-*p*. In the following, we assume *p* = 1, as it is the easiest case for experimental implementation. In the Ti^12+^ active medium the condition $\overline{\omega }_{tr}^{(y)} - \overline{\omega }_{tr}^{(z)} = 2\Omega$ corresponds to $P_{\Omega }^{\left( z \right)} \approx 12.57$ and $P_{\Omega }^{\left( y \right)} \approx 13.57$.

**Figure 3:**
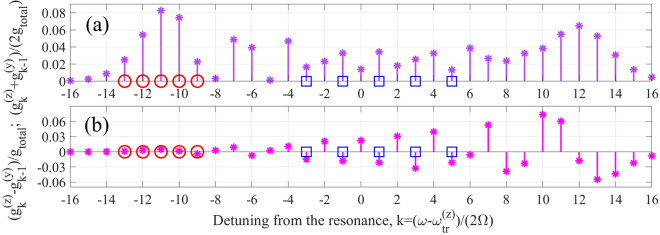
Half-sum (**a**) and difference (**b**) of the effective gain factors for the XUV radiation of *z*-polarization (5a) and *y*-polarization (5b). Red circles and blue squares mark the values of detuning, assumed in Fig. 4 and Fig. 5, respectively.

The corresponding gain spectrum calculated via Eqs. (5a) and (5b) (see Fig. 3) contains two different regions. On its left wing, particularly for *k* = − 9 to − 13 (red circles in Fig. 3), the gain coefficients are nearly the same for *z*- and *y*-polarization components of the XUV field, which allows to amplify multiple harmonics preserving their polarization state. On the other hand, in the center of the spectrum, particularly for *k* = − 3, − 1, 1, 3, and 5 (blue squares in Fig. 3), *y*-polarization component of the XUV field is amplified stronger than *z*-polarization, which allows changing the polarization state of harmonics during their amplification.

## Numerical results

In the following, we present the numerical results for the amplification of XUV pulse trains, formed by HHs of the modulating field, resonant to these two sets of the gain lines. We consider the IR field with wavelength 3.9 μm and intensity 8.26 × 10^16^ W/cm^2^, which can be produced via optical parametrical chirped pulse amplification (OPCPA)^39,40^. Such an IR field corresponds to $\overline{\omega }_{tr}^{(y)} - \overline{\omega }_{tr}^{(z)} = 2\Omega$. It is worth to note that the IR field parameters are borderline for appearance of the relativistic effects and these effects should not play yet a significant role. For example, the relativistic increase in the mass of electrons is only by 20% (the Lorentz factor is $\gamma = 1.2$). Such an increase results in 10% decrease in the electron plasma frequency and makes the plasma a little bit more transparent for the IR field. It is a favorable, although small effect. Another possible relativistic effect is an electron pileup creation by the ponderomotive force at the leading edge of the IR pulse^41,42^, which potentially could lead to a partial IR field reflection. But it could occur only if the leading front was steep enough, which is not the case for the few-picosecond IR-field pulse, considered in the present paper. The free electron and Ti^12+^ ion densities in the active medium are *N*_*e*_ = 5 × 10^19^ cm^−3^ and *N*_*ion*_ = 4.2 × 10^18^ cm^−3^, respectively. The small signal gain in the absence of the IR field is 70 cm^−1^. The coherence lifetime, ${1 \mathord{\left/ {\vphantom {1 {\gamma_{tr} }}} \right. \kern-\nulldelimiterspace} {\gamma_{tr} }}$, is nearly equal to the collision time, ${1 \mathord{\left/ {\vphantom {1 {\gamma_{Coll} }}} \right. \kern-\nulldelimiterspace} {\gamma_{Coll} }} \approx 200\;{\text{fs}}$. The typical electron temperature in the pre-created active plasma medium is 0.4–0.6 keV. However, the IR field additionally heats the plasma leading to an estimate of the characteristic absorption length for the modulating field in the range from a few millimeters to a few centimeters. Besides, the problem of the IR field absorption can be bypassed if this field is injected into the medium at the grazing incidence and focused into a line along the entire plasma channel. It is worth to note that the IR field frequency, $\Omega = 4.8 \times 10^{14} \,{\text{s}}^{ - 1}$, is close to the plasma frequency of the active medium with the relativistic correction, $\omega_{pl}^{(rel)} = {{\omega_{pl} } \mathord{\left/ {\vphantom {{\omega_{pl} } {\sqrt \gamma }}} \right. \kern-\nulldelimiterspace} {\sqrt \gamma }} = 3.6 \times 10^{14} \,{\text{s}}^{ - 1}$. However, since we consider rather long (a few picosecond) IR pulses (the IR field is effectively monochromatic), the transient effects at the front edge of the pulse can be neglected. The radiative decay rates from the states |1〉 and |2〉–|4〉 are ${1 \mathord{\left/ {\vphantom {1 {\Gamma_{rad}^{(1)} }}} \right. \kern-\nulldelimiterspace} {\Gamma_{rad}^{(1)} }} \approx 50.2\,{\text{ps}}$ and ${1 \mathord{\left/ {\vphantom {1 {\Gamma_{rad}^{(2,3,4)} }}} \right. \kern-\nulldelimiterspace} {\Gamma_{rad}^{(2,3,4)} }} \approx 3.34\,{\text{ps}}$, respectively. Further, we plot the time dependencies of the intensities of the polarization components, $I_{z,y} = \frac{c}{8\pi }\left| {\tilde{E}_{z,y} } \right|^{2}$, where $\tilde{E}_{y}$ and $\tilde{E}_{z}$ are the slowly varying amplitudes of the total harmonic field, as well as the ellipticity, which in the considered case of π/2 phase shift between the polarization components is defined as $\sigma = {{\left| {\tilde{E}_{y} } \right|^{2} } \mathord{\left/ {\vphantom {{\left| {\tilde{E}_{y} } \right|^{2} } {\left| {\tilde{E}_{z} } \right|^{2} }}} \right. \kern-\nulldelimiterspace} {\left| {\tilde{E}_{z} } \right|^{2} }}$, if $\left| {\tilde{E}_{y} } \right|^{2} \le \left| {\tilde{E}_{z} } \right|^{2}$, and $\sigma = {{\left| {\tilde{E}_{z} } \right|^{2} } \mathord{\left/ {\vphantom {{\left| {\tilde{E}_{z} } \right|^{2} } {\left| {\tilde{E}_{y} } \right|^{2} }}} \right. \kern-\nulldelimiterspace} {\left| {\tilde{E}_{y} } \right|^{2} }}$ otherwise.

**Figure 4:**
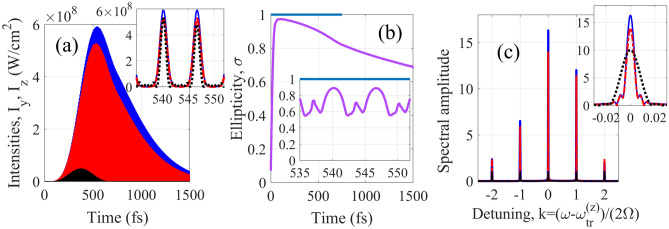
The result of amplification of the HHs resonant to the gain lines marked by red circles in Fig. 3. (**a**) Time dependences of the intensities of the polarization components of the HH field. Black dotted curve and lowest intensity correspond to the incident field. Red dash-dot and blue solid curves are *y*- and *z*- components of the amplified field, respectively. In the inset, the intensity of the incident field is multiplied by factor 30 for visibility. (**b**) Ellipticity of the incident, blue line, and amplified, lavender curve, HH field at the maxima of individual pulses. Inset shows the ellipticity time dependence within the IR-field cycle. (**c**) Fourier transform of the polarization components of the incident field (black dotted curve) and amplified field (red dash-dot and blue solid curves showing *y*-and *z*-polarization components, respectively). Inset shows the shape of individual harmonic line (in the inset, the spectrum of the incident field is multiplied by factor 10 for visibility).

Figure 4 shows the results for amplification of a train of circularly polarized pulses (the ellipticity σ = 1 at *x* = 0) formed by a combination of 129th, 131st, 133rd, 135th, and 137th harmonics of the modulating field (*k* = − 13, − 12, − 11, − 10, and − 9, respectively). The pulse duration at the entrance to the medium is 1.2 fs, the pulse repetition period is 6.5 fs, the envelope of the pulse train has a FWHM duration 270 fs, and the central wavelength is 29.33 nm (the shorter pulses can be amplified with shorter wavelength and higher intensity of the modulating field). After propagation through the active medium with the length *L* = 1 cm, the peak intensity and the total energy of the pulse train grow by 11.2 and 35.5 times, respectively. The pulse train is elongated (the bandwidth of each harmonic is reduced) due to amplification in optically dense medium, while the duration of each individual pulse is slightly increased due to nonuniform amplification of the harmonics of different orders. At the same time, the ellipticity of the XUV field is nearly preserved during the amplification process: σ = 0.89 at the peaks of the most intense pulses from the amplified pulse train at *τ* ≈ 540 fs. The ellipticity variation within the modulation cycle is caused by the difference in ellipticity of the harmonics of different orders, while the overall reduction of ellipticity with increasing time (and increasing length of the medium) is due to different gain for *z-* and *y-*polarization components of the HH field.

**Figure 5:**
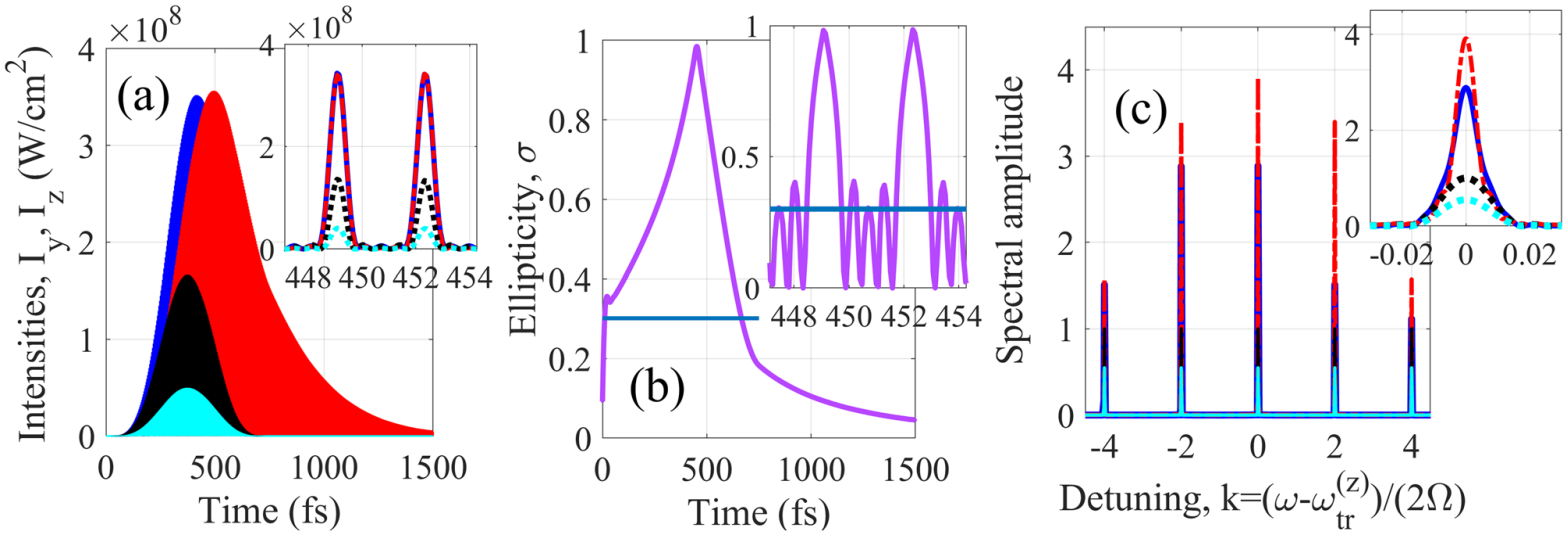
Same as in Fig. 4 but for the HHs resonant to the gain lines marked by blue squares in Fig. 3. Black and cyan dots correspond to *z*- and *y*- components of the incident field, respectively. In the insets in (**a**), (**b**) and (**c**) the intensity and Fourier transform of the incident field are not zoomed.

Figure 5 illustrates the possibility to increase ellipticity of a train of sub-femtosecond pulses formed by a combination of the 149th, 153rd, 157th, 161st, and 165th harmonics of the modulating field (*k* = − 3, − 1, 1, 3, and 5 in Fig. 3) during their amplification. In this case, the pulse duration at the entrance to the medium is 590 as, the pulse repetition period is 3.25 fs, the envelope has the same duration 270 fs, and the central wavelength is 24.85 nm. The field ellipticity at the entrance to the medium is σ(*x* = 0, *τ*) = 0.3. During the propagation through the medium, *y*-polarization component of the harmonic field grows faster than *z*-polarization, so that at *L* = 1.1 cm their peak intensities are nearly equalized (while the phase difference between them remains unchanged). As a result, at the peak of the amplified pulse train (at *τ* ≈ 450 fs) the ellipticity reaches σ = 0.995, which corresponds to almost circularly polarized radiation. At the same time, the ellipticity is nonuniform across the amplified pulse train: at the initial moments of time, *y*-polarization component is weaker than *z*-polarization, since some time is needed to establish the gain. At the peak of the amplified pulse train at *τ*≈450 fs, we have $\left| {\tilde{E}_{y} } \right|^{2} \approx \left| {\tilde{E}_{z} } \right|^{2}$, while at its tail, *y*-polarization component dominates over *z*-polarization due to stretching in time caused by its stronger amplification. The increase in ellipticity of the pulse train is accompanied by increase in its total energy by 5.1 times. Similarly to Fig. 4, each harmonic spectral line is narrowed (and the pulse train is stretched in time) due to high optical density of the medium, while the duration of sub-femtosecond pulses is slightly increased because of nonuniform amplification of the harmonics of different orders. The ellipticity of the amplified signal varies in time both on the scale of the XUV radiation envelope and within the IR field cycle. However, in the most energetic part of the pulse train the ellipticity is maximized at the peak of each sub-femtosecond pulse. Noteworthy, both in Figs. 4 and 5 the amplification nearly preserves the pulse shapes.

## Conclusion

In conclusion, in this paper we suggest an approach for the amplification of elliptically and circularly polarized sub-femtosecond XUV radiation pulses formed by HHs of an IR laser field, and increasing their ellipticity. It is proposed to use the active medium of neon-like Ti^12+^ plasma-based X-ray laser, simultaneously irradiated by the elliptically polarized HH field and the fundamental-frequency linearly polarized IR field. Due to the quadratic Stark shift caused by the IR field, the resonant energy levels of the ions oscillate in time and space with twice the IR field frequency, which results in redistribution of the gain of the active medium from the frequency of the inverted transition to the sidebands, separated from the resonance by even multiples of the modulation frequency. The gain redistribution occurs for both polarization components of the XUV radiation, parallel and perpendicular to the polarization of the IR field. With a proper choice of the intensity of the modulating field, the gain spectra for the orthogonal polarization components of the XUV radiation overlap, leading to amplification of the multifrequency elliptically polarized HH fields. The aforementioned matching of the gain spectra occurs, in particular, in a modulating field with a wavelength of 3.9 μm and an intensity of 8.26 × 10^16^ W/cm^2^. An important feature of the proposed method is its ability to amplify a set of harmonics while preserving their relative phases and approximately maintaining the temporal structure of the amplified signal. In particular, the possibility to amplify a train of circularly polarized pulses with a central wavelength of ~ 29 nm and an individual pulse duration of 1.2 fs is shown, while maintaining the polarization state and increasing the radiation energy by a factor of about 35. It is also shown that it is possible to amplify a train of pulses with a central wavelength of ~ 25 nm and a duration of 590 as with an increase in the ellipticity of radiation by more than 3 times (which corresponds to the transformation of elliptically polarized field into the circularly polarized one). In this case, the radiation energy increases by 5.1 times. The proposed method can be extended to other neon-like ions. Besides, due to the similarity of the energy structure of nickel-like and neon-like ions, this method can also be applied to the case of nickel-like active media (in particular, those based on Mo^14+^, Ag^19+^ or Kr^8+^ ions). In prospect, this opens up the possibility of amplifying the radiation of harmonics of elliptical and circular polarization in shorter-wavelength spectral ranges^43^. To the best of our knowledge, this is the first approach which suggests the amplification of sub-femtosecond pulses of XUV radiation with arbitrary elliptical polarization. Also, this is the fundamentally new approach for increasing the ellipticity of the XUV radiation pulses, which is accompanied by their energy increase rather than the energy losses inherent to the previously suggested methods. Producing of intense attosecond XUV pulses with a well-defined circular or elliptical polarization using the table-top setup would significantly broaden their applications for study of magnetic and chiral nanostructured media and ultrafast processes in them, including the dynamical nanoscale imaging and coherent control of the ultrafast spin flip processes.

## Supplementary Information

Supplementary Information.
