# Supplementary material for: Amplification of elliptically polarized sub-femtosecond pulses in neon-like X-ray laser modulated by an IR field
Source: Sci Rep. 2022 Apr 13;12:6204. doi: 10.1038/s41598-022-09701-z (PMC9008065; doi:10.1038/s41598-022-09701-z)
Supplement: Supplementary file 1 — Supplementary Information. [file 41598_2022_9701_MOESM1_ESM.pdf]

Amplification of Elliptically Polarized Sub-Femtosecond Pulses in Neon-Like X-Ray Laser Modulated by an IR Field: Supplementary Information

I. Theoretical model

Propagation of the XUV radiation through the medium is described by the one-dimensional wave equation

$$\frac{\partial^2 \vec{E}}{\partial x^2} - \frac{\varepsilon_{pl}^{(XUV)}}{c^2}\frac{\partial^2 \vec{E}}{\partial t^2} = \frac{4\pi}{c}\frac{\partial^2 \vec{P}}{\partial t^2}, \tag{S1}$$

where $\vec{E}$ is the electric field strength of the HH field, $\varepsilon_{pl}^{(XUV)} = 1 - \omega_{pl}^2 / \omega^2$ is the dielectric constant of the plasma for the XUV radiation, $\omega_{pl} = \sqrt{4\pi N_e e^2 / m_e}$ is the electron plasma frequency, N_e is the concentration of free electrons in the plasma, e and m_e are the charge and mass of the electron, respectively, c is the speed of light in vacuum, and $\vec{P}$ is the resonant polarization of the medium, which is determined by the density matrix elements of the Ti^{12+} ions as

$$\vec{P} = N_{ion}\left(\rho_{12}\vec{d}_{21} + \rho_{13}\vec{d}_{31} + \rho_{14}\vec{d}_{41}\right) + \text{c.c.} \tag{S2}$$

In Eq. (S2) N_{ion} is the density of Ti^{12+} ions in the $|1\rangle$, $|2\rangle$, $|3\rangle$, and $|4\rangle$ states at the initial instant of time, whereas $\vec{d}_{i1}$ are the dipole moments of transitions $|i\rangle \leftrightarrow |1\rangle$, i=2,3,4, and ρ_{1i} are the quantum coherences (off-diagonal elements of the density matrix) at these transitions. The evolution of the quantum state of Ti^{12+} ions under the influence of XUV radiation is described by the density matrix equations

$$\begin{cases} \dfrac{\partial \rho_{11}}{\partial t} + \gamma_{11}\rho_{11} = \dfrac{i}{\hbar}\sum_{s=1}^{4}\left(\rho_{s1}\vec{d}_{1s} - \rho_{1s}\vec{d}_{s1}\right)\vec{E}, \\ \dfrac{\partial \rho_{ii}}{\partial t} + \gamma_{ii}\rho_{ii} = A\rho_{11} + \dfrac{i}{\hbar}\sum_{s=1}^{4}\left(\rho_{si}\vec{d}_{is} - \rho_{is}\vec{d}_{si}\right)\vec{E}, \text{ if } i \neq 1, \\ \dfrac{\partial \rho_{ij}}{\partial t} + \left(i\omega_{ij}(t,x) + \gamma_{ij}\right)\rho_{ij} = \dfrac{i}{\hbar}\sum_{s=1}^{4}\left(\rho_{sj}\vec{d}_{is} - \rho_{is}\vec{d}_{sj}\right)\vec{E}, \text{ if } j \neq i, \end{cases} \tag{S3}$$

where ρ_{ij} are the density matrix elements, $\omega_{ij}(t,x)$ are the frequencies of quantum transitions $|i\rangle \leftrightarrow |j\rangle$ given by Eqs. (4) of the main text, $\hbar$ is the Planck's constant, A is the rate of spontaneous radiative transition from the $|1\rangle$ state to each of the $|2\rangle$, $|3\rangle$, and $|4\rangle$ states, $1/A$ = 242.5 ps [1], and γ_{ij} are the relaxation rates of the density-matrix elements, which are determined as follows. The relaxation rate of the density matrix diagonal element ρ_{ii} is the sum of the rates of radiative transitions from the $|i\rangle$ state to all lower-energy ionic states, $\Gamma_{rad}^{(i)}$, and the rate of tunneling ionization from the $|i\rangle$ state under the influence of the IR field, $w_{ion}^{(i)}$ (which is calculated using the Perelomov-Popov-Terent'ev formula [2]), i.e. $\gamma_{ii} = \Gamma_{rad}^{(i)} + w_{ion}^{(i)}$. Note that in the range of IR intensities under consideration, ionization from resonant states is not significant, $w_{ion}^{(i)} \ll \Gamma_{rad}^{(i)}\ (\forall i)$. The relaxation rate of an off-diagonal element ρ_{ij} of the density matrix is determined as $\gamma_{ij} = (\gamma_{ii}+\gamma_{ij})/2+\gamma_{Coll}$, where γ_{Coll} is the frequency of collisions in the plasma.

Next, we turn to the local time $t \to \tau = t - x\sqrt{\varepsilon_{pl}^{(XUV)}}\Big/c$ and look for a solution of the system of equations (S1)-(S3) using the approximation of slowly varying amplitudes, i.e. assuming that

$$\begin{aligned}
&\vec{F}(x,\tau) = \frac{1}{2}\left(\vec{z}_0\tilde{F}_z(x,\tau) + \vec{y}_0\tilde{F}_y(x,\tau)\right)\exp\left(-i\omega\tau\right) + \text{c.c., where } F \text{ stands for } E \text{ or } P,\\
&\rho_{12}\left(x,\tau\right) = \tilde{\rho}_{12}\left(x,\tau\right)e^{-i\omega\tau},\ \rho_{13}\left(x,\tau\right) = \tilde{\rho}_{13}\left(x,\tau\right)e^{-i\omega\tau},\ \rho_{14}\left(x,\tau\right) = \tilde{\rho}_{14}\left(x,\tau\right)e^{-i\omega\tau},\\
&\rho_{ij} = \rho_{ji}^{*}\ \textit{and}\ \rho_{ij}\left(x,\tau\right) = \tilde{\rho}_{ij}\left(x,\tau\right)\ \textit{if}\ ij \neq \left\{12, 21, 13, 31, 14, 41\right\}.
\end{aligned} \tag{S4}$$

Here $\tilde{F}_z(x,\tau)$ and $\tilde{F}_y(x,\tau)$ stand for slowly varying complex amplitudes of the polarization components of either the XUV radiation or the resonant polarization of the medium, $\left|\partial\tilde{F}_{z,y}/\partial\tau\right| \ll \omega\left|\tilde{F}_{z,y}\right|$ and $\left|\partial\tilde{E}_{z,y}/\partial x\right| \ll \omega\sqrt{\varepsilon_{pl}^{(XUV)}}\left|\tilde{E}_{z,y}\right|\Big/c$, whereas $\tilde{\rho}_{ij}\left(x,\tau\right)$ are slowly varying amplitudes of the density-matrix elements of the medium, $\left|\partial\tilde{\rho}_{ij}/\partial\tau\right| \ll \omega\left|\tilde{\rho}_{ij}\right|$ and $\left|\partial\tilde{\rho}_{ij}/\partial x\right| \ll \omega\sqrt{\varepsilon_{pl}^{(XUV)}}\left|\tilde{\rho}_{ij}\right|\Big/c$. With this substitution, the wave equation for the HH field turns to

$$\frac{\partial\vec{\tilde{E}}}{\partial x} = i2\pi\frac{\omega}{c\sqrt{\varepsilon_{pl}^{(XUV)}}}\vec{\tilde{P}}, \tag{S5}$$

while the density matrix equations take the form

$$\begin{cases}
\dfrac{\partial\tilde{\rho}_{11}}{\partial\tau} + \gamma_{11}\tilde{\rho}_{11} = \dfrac{i}{2\hbar}d_z\left(\tilde{\rho}_{12}^{*}\tilde{E}_z - \tilde{\rho}_{12}\tilde{E}_z^{*}\right) - \dfrac{1}{2\hbar}d_y\left(\tilde{\rho}_{13}^{*}\tilde{E}_y + \tilde{\rho}_{13}\tilde{E}_y^{*}\right) - \dfrac{1}{2\hbar}d_y\left(\tilde{\rho}_{14}^{*}\tilde{E}_y + \tilde{\rho}_{14}\tilde{E}_y^{*}\right),\\
\dfrac{\partial\tilde{\rho}_{22}}{\partial\tau} + \gamma_{22}\tilde{\rho}_{22} = A\tilde{\rho}_{11} - \dfrac{i}{2\hbar}d_z\left(\tilde{\rho}_{12}^{*}\tilde{E}_z - \tilde{\rho}_{12}\tilde{E}_z^{*}\right),\\
\dfrac{\partial\tilde{\rho}_{33}}{\partial\tau} + \gamma_{33}\tilde{\rho}_{33} = A\tilde{\rho}_{11} + \dfrac{1}{2\hbar}d_y\left(\tilde{\rho}_{13}^{*}\tilde{E}_y + \tilde{\rho}_{13}\tilde{E}_y^{*}\right),\\
\dfrac{\partial\tilde{\rho}_{44}}{\partial\tau} + \gamma_{44}\tilde{\rho}_{44} = A\tilde{\rho}_{11} + \dfrac{1}{2\hbar}d_y\left(\tilde{\rho}_{14}^{*}\tilde{E}_y + \tilde{\rho}_{14}\tilde{E}_y^{*}\right),\\
\dfrac{\partial\tilde{\rho}_{12}}{\partial\tau} + \left[i\left(\omega_{12}\left(\tau,x\right) - \omega\right) + \gamma_{12}\right]\tilde{\rho}_{12} = -\dfrac{i}{2\hbar}d_z\left(\tilde{\rho}_{11} - \tilde{\rho}_{22}\right)\tilde{E}_z - \dfrac{1}{2\hbar}d_y\tilde{\rho}_{23}^{*}\tilde{E}_y - \dfrac{1}{2\hbar}d_y\tilde{\rho}_{24}^{*}\tilde{E}_y,\\
\dfrac{\partial\tilde{\rho}_{13}}{\partial\tau} + \left[i\left(\omega_{13}\left(\tau,x\right) - \omega\right) + \gamma_{13}\right]\tilde{\rho}_{13} = \dfrac{1}{2\hbar}d_y\left(\tilde{\rho}_{11} - \tilde{\rho}_{33}\right)\tilde{E}_y + \dfrac{i}{2\hbar}d_z\tilde{\rho}_{23}\tilde{E}_z - \dfrac{1}{2\hbar}d_y\tilde{\rho}_{34}^{*}\tilde{E}_y,\\
\dfrac{\partial\tilde{\rho}_{14}}{\partial\tau} + \left[i\left(\omega_{14}\left(\tau,x\right) - \omega\right) + \gamma_{14}\right]\tilde{\rho}_{14} = \dfrac{1}{2\hbar}d_y\left(\tilde{\rho}_{11} - \tilde{\rho}_{44}\right)\tilde{E}_y + \dfrac{i}{2\hbar}d_z\tilde{\rho}_{24}\tilde{E}_z - \dfrac{1}{2\hbar}d_y\tilde{\rho}_{34}\tilde{E}_y,\\
\dfrac{\partial\tilde{\rho}_{23}}{\partial\tau} + \left[i\omega_{23}\left(\tau,x\right) + \gamma_{23}\right]\tilde{\rho}_{23} = \dfrac{i}{2\hbar}d_z\tilde{\rho}_{13}\tilde{E}_z^{*} + \dfrac{1}{2\hbar}d_y\tilde{\rho}_{12}^{*}\tilde{E}_y,\\
\dfrac{\partial\tilde{\rho}_{24}}{\partial\tau} + \left[i\omega_{24}\left(\tau,x\right) + \gamma_{24}\right]\tilde{\rho}_{24} = \dfrac{i}{2\hbar}d_z\tilde{\rho}_{14}\tilde{E}_z^{*} + \dfrac{1}{2\hbar}d_y\tilde{\rho}_{12}^{*}\tilde{E}_y,\\
\dfrac{\partial\tilde{\rho}_{34}}{\partial\tau} + \left[i\omega_{34}\left(\tau,x\right) + \gamma_{34}\right]\tilde{\rho}_{34} = \dfrac{1}{2\hbar}d_y\tilde{\rho}_{14}\tilde{E}_y^{*} + \dfrac{1}{2\hbar}d_y\tilde{\rho}_{13}^{*}\tilde{E}_y,
\end{cases} \tag{S6}$$

where $d_z = D\big/\sqrt{3}$ and $d_y = D\big/\sqrt{6}$, whereas $D \equiv \left|\left\langle 3p^1S_0 \,\|\, D \,\|\, 3s^1P_1\right\rangle\right|$ is the reduced matrix element of dipole moment of the inverted transition $3p^1S_0 \leftrightarrow 3s^1P_1$. In Eqs. (S6) we also assume that the HH field is nearly resonant to the transitions $|1\rangle \leftrightarrow |2\rangle$ and $|1\rangle \leftrightarrow |3\rangle,|4\rangle$: $\left|\omega - \bar{\omega}_{tr}^{(z)}\right| \ll \omega$ and $\left|\omega - \bar{\omega}_{tr}^{(y)}\right| \ll \omega$, and use the rotating wave approximation.

Equations (S5) and (S6) were solved numerically, assuming that (i) there is no reflections from the boundaries of the medium, so that $\vec{E}(x=0,\tau)=\vec{E}^{(inc)}(t)$; (ii) at $\tau=0$ among the states |1⟩- |4⟩, only the state |1⟩ is populated: $\tilde{\rho}_{11}(x,\tau=0)=1$ while $\tilde{\rho}_{ii}(x,\tau=0)=0$ for $i\neq1$; and (iii) there are random initial values of the quantum coherences at the inverted |1⟩ ↔ |2⟩, |3⟩, |4⟩ transitions, responsible for simulation of the amplified spontaneous emission (ASE) of the medium (see [3-6]), whereas the initial values of the remaining coherences are equal to zero. We also note that at $\tau=0$ the XUV radiation in the medium is absent (with the exception of its front boundary): $\vec{E}(x\neq0,\tau=0)=0$.

In the numerical simulations for all the harmonics from the seeding XUV radiation, we assume the envelope

$$\begin{aligned}\tilde{E}_{z,inc}^{(k)}(\tau)&=E_0^{(z)}\left[\theta(\tau)-\theta(\tau-\tau_{zero})\right]\sin^2\left(\pi\tau/\tau_{zero}\right),\\ \tilde{E}_{y,inc}^{(k)}(\tau)&=E_0^{(y)}\left[\theta(\tau)-\theta(\tau-\tau_{zero})\right]\sin^2\left(\pi\tau/\tau_{zero}\right),\end{aligned}\tag{S7}$$

where $k_{\min}\le k\le k_{\max}$ (see Eq. (3) of the main text), $\theta(\tau)$ is the Heaviside unit step function: $\theta(\tau)=0$ for $\tau<0$ and $\theta(\tau)=1$ for $\tau\geq0$; while the parameter τ_{zero} determines the duration of the seed field envelope from zero to zero. In further calculations, we assume τ_{zero} = 750 fs. In this case, the FWHM of intensity envelope is about 270 fs. These values of the parameters were chosen from the consideration that for efficient amplification of the incident field, its duration should exceed the transient time for resonant polarization of the active medium, which in our case is about 200 fs.

II. Analytical solution

In order to derive the analytical solution given by Eqs. (5) of the main text, we made the additional assumptions. In particular, we assume that (i) during the considered time interval the populations of states don't change, $\tilde{\rho}_{ii}(x,\tau)=\tilde{\rho}_{ii}(x,\tau=0)$, i=1,2,3,4, (ii) the coherences on the dipole-forbidden transitions between the |2⟩, |3⟩, and |4⟩ states are identically equal to zero, and (iii) the coherences at the dipole-allowed |1⟩↔|2⟩, |1⟩↔|3⟩, and |1⟩↔|4⟩ transitions are equal to zero before the arrival of the XUV field, which corresponds to neglecting the spontaneous emission of the medium. In these approximations, the amplification of the polarization components of the XUV radiation occurs independently and can be analyzed separately. In such a case, *z*- and *y*-polarization components of the HH field satisfy the equations

$$\begin{cases}\dfrac{\partial\tilde{E}_z}{\partial x}=i4\pi N_{ion}d_z\dfrac{\omega}{c\sqrt{\varepsilon_{pl}^{(XUV)}}}\tilde{\rho}_{12},\\[2ex] \dfrac{\partial\tilde{\rho}_{12}}{\partial\tau}+\left\{i\left(\bar{\omega}_{tr}^{(z)}-\omega\right)+i\Delta_\Omega^{(z)}\cos\left[2\left(\Omega\tau+\Delta Kx\right)\right]+\gamma_z\right\}\tilde{\rho}_{12}=-i\dfrac{d_z\tilde{E}_z}{2\hbar},\end{cases}\tag{S8a}$$

and

$$\begin{cases}\dfrac{\partial\tilde{E}_y}{\partial x}=4\pi N_{ion}d_y\dfrac{\omega}{c\sqrt{\varepsilon_{pl}^{(XUV)}}}\left(\tilde{\rho}_{13}+\tilde{\rho}_{14}\right),\\[2ex] \dfrac{\partial\tilde{\rho}_{13}}{\partial\tau}+\left\{i\left(\bar{\omega}_{tr}^{(y)}-\omega\right)+i\Delta_\Omega^{(y)}\cos\left[2\left(\Omega\tau+\Delta Kx\right)\right]+\gamma_y\right\}\tilde{\rho}_{13}=\dfrac{d_y\tilde{E}_y}{2\hbar},\\[2ex] \dfrac{\partial\tilde{\rho}_{14}}{\partial\tau}+\left\{i\left(\bar{\omega}_{tr}^{(y)}-\omega\right)+i\Delta_\Omega^{(y)}\cos\left[2\left(\Omega\tau+\Delta Kx\right)\right]+\gamma_y\right\}\tilde{\rho}_{14}=\dfrac{d_y\tilde{E}_y}{2\hbar},\end{cases}\tag{S8b}$$

respectively, where $\gamma_z \equiv \gamma_{12}$ and $\gamma_y \equiv \gamma_{13} = \gamma_{14}$, while ΔK, $\bar{\omega}_{tr}^{(z)}$, $\bar{\omega}_{tr}^{(y)}$, $\Delta_\Omega^{(z)}$ and $\Delta_\Omega^{(y)}$ are defined in the main text.

To derive the analytical solution, we assume the instantaneous switch on of the field of each harmonic from the seeding XUV field (3): $\tilde{E}_{z,inc}^{(k)}(\tau) = \theta(\tau) E_{z,0}^{(k)}$ and $\tilde{E}_{y,inc}^{(k)}(\tau) = \theta(\tau) E_{y,0}^{(k)}$, where $E_{z,0}^{(k)}$ and $E_{y,0}^{(k)}$ are complex numbers, and look for a solution for the polarization components of the HH field in the form

$$\tilde{E}_z = \sum_{k=k_{\min}}^{k_{\max}} \tilde{E}_z^{(k)}(x,\tau)\theta(\tau)e^{-i2k\Omega\tau} \text{ and } \tilde{E}_y = \sum_{k=k_{\min}}^{k_{\max}} \tilde{E}_y^{(k)}(x,\tau)\theta(\tau)e^{-i2k\Omega\tau}, \tag{S9}$$

where $k_{\min} \le k \le k_{\max}$ (see Eq. (3) of the main text), while $\tilde{E}_z^{(k)}(x,\tau)$ and $\tilde{E}_y^{(k)}(x,\tau)$ are the slowly-varying amplitudes of polarization components of the "k-th" harmonic. At the entrance to the medium $\tilde{E}_z^{(k)}(x,\tau) = E_{z,0}^{(k)}$ and $\tilde{E}_y^{(k)}(x,\tau) = E_{y,0}^{(k)}$. We also assume that (i) the difference between the time-averaged frequencies of the transitions $|1\rangle \leftrightarrow |2\rangle$ and $|1\rangle \leftrightarrow |3\rangle,|4\rangle$ is an even multiple of the modulation frequency, $\bar{\omega}_{tr}^{(y)} - \bar{\omega}_{tr}^{(z)} = 2\Omega p$, where p is an integer, and (ii) the carrier frequency of seeding harmonic field coincides with the time-averaged frequency of the transition $|1\rangle \leftrightarrow |2\rangle$, $\omega = \bar{\omega}_{tr}^{(z)}$.

Let us start with Eqs. (S8a) and seek the solution for $\tilde{\rho}_{12}(x,\tau)$ in the form:

$$\tilde{\rho}_{12}(x,\tau) = \hat{\rho}_{12}(x,\tau)\exp\left[-\gamma_z\tau - iP_\Omega^{(z)}\sin(2\Omega\tau + 2\Delta K x)\right], \tag{S10}$$

where $P_\Omega^{(z)} = \Delta_\Omega^{(z)}/(2\Omega)$ is the modulation index of the transition $|1\rangle \leftrightarrow |2\rangle$, and $\hat{\rho}_{12}(x,\tau)$ obeys the following equation

$$\frac{\partial \hat{\rho}_{12}}{\partial \tau} = -i\frac{d_z}{2\hbar}\sum_{k=k_{\min}}^{k_{\max}}\sum_{n=-\infty}^{\infty} J_n\left(P_\Omega^{(z)}\right)\tilde{E}_z^{(k)}(x,\tau)e^{i2n\Delta K x}\theta(\tau)e^{\left[\gamma_z + i2(n-k)\Omega\right]\tau}, \tag{S11}$$

which was derived using Eq. (S9) and the Jacobi-Anger expansion, $e^{\pm ip\sin\phi} = \sum_{n=-\infty}^{\infty} J_n(p)e^{\pm in\phi}$; $J_n(x)$ is the Bessel function of the first kind of order n. Implying also the inertialess relationship between the resonant polarization of the medium and the XUV radiation (see [7]) and assuming $\Omega/\gamma_z \gg 1$, we integrate Eq. (S11) and find the following solution for $\tilde{\rho}_{12}(x,\tau)$:

$$\tilde{\rho}_{12} = -i\frac{d_z}{2\hbar\gamma_z}\theta(\tau)\left(1 - e^{-\gamma_z\tau}\right)\sum_{k=k_{\min}}^{k_{\max}}\sum_{m=-\infty}^{\infty} J_k\left(P_\Omega^{(z)}\right)J_m\left(P_\Omega^{(z)}\right)\tilde{E}_z^{(k)}(x,\tau)e^{i2(k-m)\Delta K x}e^{-i2m\Omega\tau}. \tag{S12}$$

By plugging Eq. (S12) into the first equation of (S8a) and integrating the resulting expression, we obtain the solution for the slowly varying amplitude of z-polarization component of the high-harmonic field at the exit from the medium in the form (S9), where the amplitude of z-polarization component of the "k-th" harmonic field has a form

$$\begin{cases} \tilde{E}_z^{(k)}(x,\tau) = E_{z,0}^{(k)}\theta(\tau)\exp\left[g_k^{(z)}\left(P_\Omega^{(z)},\tau\right)x\right], \\ g_k^{(z)}\left(P_\Omega^{(z)},\tau\right) = g_{total}^{(z)} J_k^2\left(P_\Omega^{(z)}\right)\left(1 - e^{-\gamma_z\tau}\right). \end{cases} \tag{S13}$$

Here $g_k^{(z)}\left(P_\Omega^{(z)},\tau\right)$ is the effective gain coefficient for z-polarization components of the HH field and $g_{total}^{(z)} = \frac{2\pi\omega N_{ion} d_z^2}{\hbar c \gamma_z \sqrt{\varepsilon_{pl}^{(XUV)}}}$ is the gain factor for z-polarization components of the resonant

XUV field in the absence of the time-dependent Stark shift. Eq. (S13) implies that the plasma is strongly dispersive for the modulating IR field, and the value $1/\Delta K$ is much smaller than the gain length for the XUV radiation. In this case, the rescattering of HHs into each other is suppressed (see [6,7]), and the harmonics of different orders in each polarization component are amplified independently.

Similarly, one obtains the following solution of Eqs. (S8b) for *y*-polarized component of "*k*-th" harmonic field:

$$\begin{cases} \tilde{E}_y^{(k)}(x,\tau) = E_{y,0}^{(k)}\theta(\tau)\exp\left[g_{k-p}^{(y)}\left(P_\Omega^{(y)},\tau\right)x\right], \\ g_{k-p}^{(y)}\left(P_\Omega^{(y)},\tau\right) = g_{total}^{(y)} J_{k-p}^2\left(P_\Omega^{(y)}\right)\left(1-e^{-\gamma_{tr}\tau}\right), \end{cases} \tag{S14}$$

where $P_\Omega^{(y)} = \Delta_\Omega^{(y)}/(2\Omega)$ is the IR field-induced frequency modulation index of the $|1\rangle \leftrightarrow |3\rangle,|4\rangle$ transitions, $g_{k-p}^{(y)}\left(P_\Omega^{(z)},\tau\right)$ is the effective gain coefficient for *y*-polarization component of the HH field, while $g_{total}^{(y)} = \dfrac{4\pi\omega N_{ion} d_y^2}{\hbar c \gamma_y \sqrt{\varepsilon_{pl}^{(XUV)}}}$ is the gain factor for *y*-polarization component of the resonant XUV field in the absence of the time-dependent Stark shift. Eqs. (S13) and (S14) coincide with Eqs. (5a,b) of the main text, if one takes into account that $\gamma_{tr} = \gamma_z \simeq \gamma_y \simeq \gamma_{Coll}$, and $d_y = d_z/\sqrt{2}$, so that $g_{total} = g_{total}^{(z)} = g_{total}^{(y)}$.

III. Relevant parameters of the resonant ions and the plasma:

Parameter	Value
Ionization potential (unperturbed) of the ground state of Ti^{12+} ions, $I_{gr}^{(0)}$	787.67 eV
Ionization potential (unperturbed) of the upper lasing state $|1\rangle$, $I_1^{(0)} = I_{gr}^{(0)} - E_1^{(0)}$	282.06 eV
Ionization potential (unperturbed) of the lower lasing states $|2\rangle$, $|3\rangle$, $|4\rangle$, $I_{2-4}^{(0)} = I_{gr}^{(0)} - E_{2-4}^{(0)}$, where $E_{2-4}^{(0)} \equiv E_2^{(0)} = E_3^{(0)} = E_4^{(0)}$	323.33 eV
The amplitude of quadratic Stark shift of the state $|1\rangle$, $\Delta_E^{(1)}$	1.0266 eV for $I_{IR} = 8.26\times10^{16}$ W/cm^2
The amplitude of quadratic Stark shift of the state $|2\rangle$, $\Delta_E^{(2)}$	-6.9606 eV for $I_{IR} = 8.26\times10^{16}$ W/cm^2
The amplitude of quadratic Stark shift of the states $|3\rangle$ and $|4\rangle$, $\Delta_E^{(3)} = \Delta_E^{(4)}$	-7.5960 eV for $I_{IR} = 8.26\times10^{16}$ W/cm^2
Radioactive decay rate from the state $|1\rangle$, $\Gamma_{rad}^{(1)}$	1.9941×10^{10} 1/s
Radioactive decay rates from the states $|2\rangle$-$|4\rangle$, $\Gamma_{rad}^{(2)} = \Gamma_{rad}^{(3)} = \Gamma_{rad}^{(4)}$	2.9979×10^{11} 1/s
Ionization rate from the state $|1\rangle$, $w_{ion}^{(1)}$	8.0789×10^{9} 1/s for

	$I_{IR} = 8.26\times10^{16}$ W/cm^2
Ionization rate from the state $\lvert 2\rangle$, $w_{ion}^{(2)}$	3.3750×10^{6} 1/s for $I_{IR} = 8.26\times10^{16}$ W/cm^2
Ionization rates from the states $\lvert 3\rangle$ and $\lvert 4\rangle$, $w_{ion}^{(3)} = w_{ion}^{(4)}$	2.2370×10^{4} 1/s for $I_{IR} = 8.26\times10^{16}$ W/cm^2
The frequency of collisions in the plasma, γ_{Coll}	4.6972×10^{12} 1/s
The rate of spontaneous radiative transition from the $\lvert 1\rangle$ state to each of the $\lvert 2\rangle$, $\lvert 3\rangle$, and $\lvert 4\rangle$ states, A	4.1233×10^{9} 1/s
The reduced matrix element of dipole moment of the transition $3p\,^1S_0 \leftrightarrow 3s\,^1P_1$, D	1.0338 D

References

1. Gu, M.F. The flexible atomic code. *Canadian Journal of Physics.* **86**, 675-689 (2008).
2. Popov, V.S. Tunnel and multiphoton ionization of atoms and ions in a strong laser field (Keldysh theory. Physics – Uspekhi. **47** (9), 855-885 (2004).
3. Glauber, R. and Haake, F. The initiation of superfluorescence. *Phys. Lett. A*. **68**, 29-32 (1978).
4. Haake, F., et al. Macroscopic quantum fluctuations in superfluorescence. *Phys. Rev. Lett*. **42**, 1740-1743 (1979).
5. Gross, M. and Haroche, S. Superradiance: An essay on the theory of collective spontaneous emission. *Phys. Reports* **93**, 301-396 (1982).
6. Antonov, V.A., Han, K.Ch., Akhmedzhanov, T.R., Scully, M., and Kocharovskaya, O. Attosecond pulse amplification in a plasma-based x-ray laser dressed by an infrared laser field. *Phys. Rev. Lett.* **123**, 243903 (2019).
7. Khairulin, I.R., Antonov, V.A., Ryabikin, M.Yu., and Kocharovskaya, O. Sub-fs pulse formation in a seeded hydrogenlike plasma-based x-ray laser dressed by an infrared field: Analytical theory and numerical optimization. *Phys. Rev. Research*. **2**, 023255 (2020).
